# Use of CRISPRoff and synthetic Notch to modulate and relay endogenous gene expression programs in engineered cells

**DOI:** 10.3389/fbioe.2024.1346810

**Published:** 2024-06-18

**Authors:** Shuqun Shi, Catherine A. Hamann, Joanne C. Lee, Jonathan M. Brunger

**Affiliations:** ^1^ Department of Biomedical Engineering, Vanderbilt University, Nashville, TN, United States; ^2^ Center for Stem Cell Biology, Vanderbilt University, Nashville, TN, United States

**Keywords:** synthetic biology, gene regulation, gene editing, epigenetic editing, stem cell technology, designer cells, synNotch, CRISPRoff

## Abstract

Uncovering the stimulus-response histories that give rise to cell fates and behaviors is an area of great interest in developmental biology, tissue engineering, and regenerative medicine. A comprehensive accounting of cell experiences that lead to the development of organs and tissues can help us to understand developmental anomalies that may underly disease. Perhaps more provocatively, such a record can also reveal clues as to how to drive cell collective decision-making processes, which may yield predictable cell-based therapies or facilitate production of tissue substitutes for transplantation or *in vitro* screening of prospective therapies to mitigate disease. Toward this end, various methods have been applied to molecularly trace developmental trajectories and record interaction histories of cells. Typical methods involve artificial gene circuits based on recombinases that activate a suite of fluorescent reporters or CRISPR-Cas9 genome writing technologies whose nucleic acid-based record keeping serves to chronicle cell-cell interactions or past exposure to stimuli of interests. Exciting expansions of the synthetic biology toolkit with artificial receptors that permit establishment of defined input-to-output linkages of cell decision-making processes opens the door to not only record cell-cell interactions, but to also potentiate directed manipulation of the outcomes of such interactions via regulation of carefully selected transgenes. Here, we combine CRISPR-based strategies to genetically and epigenetically manipulate cells to express components of the synthetic Notch receptor platform, a widely used artificial cell signaling module. Our approach gives rise to the ability to conditionally record interactions between human cells, where the record of engagement depends on expression of a state-specific marker of a subset of cells in a population. Further, such signal-competent interactions can be used to direct differentiation of human embryonic stem cells toward pre-selected fates based on assigned synNotch outputs. We also implemented CRISPR-based manipulation of native gene expression profiles to bias outcomes of cell engagement histories in a targeted manner. Thus, we present a useful strategy that gives rise to both state-specific recording of cell-cell interactions as well as methods to intentionally influence products of such cell-cell exchanges.

## 1 Introduction

Cell-cell contact and communication are ubiquitous in multicellular organisms. They are essential in fundamental biological processes, including development, neuronal and immune responses, stem cell fate allocation, and tumorigenesis (Frieda et al., 2017; Bechtel et al., 2021). Various methods have been established to molecularly trace developmental trajectories and record interaction histories of cells within a niche. Generally, these technologies involve artificial gene circuits that yield reporter protein expression (e.g., Brainbow (Livet et al., 2007)) or archival of cellular responses to inputs via CRISPR-based scratchpads that can be read via single-cell sequencing and computationally mapped to reveal transaction logs of cells (e.g., MEMOIR, CAMERA, mSCRIBE) (Frieda et al., 2017; He et al., 2017; Tang and Liu, 2018). While powerful for unveiling the flow of information between cells and their niche, these methods do not enable facile, direct perturbation of cellular responses to selected inputs (He and Perrimon, 2023). Such limitations restrict usage of these tools to records of cellular interactions and do not offer a means to readily interrogate how tunable changes in local signal strength of developmental cues may give rise to differential morphogenetic outcomes.

One tool that has recently been deployed to record contact histories of cells in developing mouse embryos is the synthetic Notch (synNotch) receptor platform (Zhang et al., 2022). SynNotch is based on the native Notch signaling apparatus (Morsut et al., 2016; Toda et al., 2020). In this system, the ligand-binding domain of Notch is replaced with an extracellular recognition motif borrowed from nanobodies or single chain variable fragments (scFvs) derived from monoclonal antibodies. Such affinity motifs allow for programmable recognition of ligands of choice by synNotch receptors. Further, in the synNotch system, the Notch intracellular domain is replaced by an artificial transcription factor such as the tetracycline transactivator. Thus, when synNotch receptors bind an immobilized ligand (i.e., presented on neighboring cells or anchored to the extracellular environment), the resultant mechanical force exposes protease intramembrane cleavage sites in the Notch core, potentiating release of the intracellular transcription factor. This allows for subsequent expression of user-specified target transgenes (Malaguti et al., 2022; Lee et al., 2023). In this way, synNotch activation can tune defined cellular responses to selected inputs. Thus, one can design synthetic cell-cell communication programs using synNotch circuits, where synNotch ligand-presenting “sender” cells activate transgene expression in synNotch receptor-competent “receiver” cells.

As previously deployed in developmental tracking studies, a GFP-sensitive synNotch receptor activated expression of reporter transgenes to record contact histories between lineage-specific, GFP ligand-expressing sender cells and synNotch receiver cells (Zhang et al., 2022). This approach allowed for exquisite tracing of prior contact between cells originating in certain organs (such as the endothelium of the developing heart) early in embryogenesis and subsequently migrating to occupy other organs later in development (e.g., to establish vasculature in the liver). Inspired by this, we sought to port this system into human cells and elaborate upon it. Our goals were to extend this methodology to not only trace cellular interactions via fluorescent protein expression, but to also gate expression of synNotch ligand based on native levels of markers of cell state, such as endogenous transcription factor expression levels. Critically, we also sought to leverage the flexible nature of synNotch to direct cell fate specification based on synNotch activation strength in engineered receiver pluripotent stem cells. Finally, we exploited CRISPR-based repression systems to regulate levels of ligand expressed by synNotch sender cells and demonstrate that such tools, which enable physiologic tuning of native gene expression levels, meaningfully perturb fate specification of synNotch receiver stem cells.

PAX6 is a transcription factor that serves as a marker of differentiation in several cell types, including neural progenitors (Davis et al., 2008; Davis et al., 2009). In the rapidly growing area of brain organoid production, induction of PAX6 serves as a marker to indicate successful transition from pluripotency to neuroectodermal tissue, and these cells give rise to several further specialized neuronal subtypes in developing organoids (Cederquist et al., 2018). Changes in PAX6 dosage caused by genomic alteration result in eye malformation and central nervous system defects (Glaser et al., 1994; Schedl et al., 1996), indicating the relevance of PAX6 levels in the context of human development. PAX6 is also expressed in HEK293T cells, which are known to express markers of not only renal progenitors and adrenal gland, but also of neuronal-specific genes. Here, we gated expression of the synNotch ligand GFP (GFPL) on PAX6 levels by performing nuclease-mediated targeted addition of GFPL transgene to the *PAX6* locus in HEK293T cells. We also used CRISPR-Cas9 to engineer HEK293 synNotch receiver cells via targeted integration to the AAVSI safe harbor locus. Monitoring fluorescent protein expression in this system enables tracking of cell-cell interaction histories. Further, we developed a CRISPRoff toolkit to heritably suppress *PAX6* in sender cells, and then deployed these cells to regulate expression of the differentiation factor Ngn2 in human embryonic stem cells (hESCs). Results illustrate the potential of combining CRISPR-based technologies with the synNotch receptor platform to both track cell-cell interactions and bias cell state transitions based on manipulable, endogenous regulation of synNotch platform components.

## 2 Materials and methods

### 2.1 Generation of knock-in cell lines

#### 2.1.1 Generation of GFP ligand knock-in to the *PAX6* locus of HEK293T cells

To produce the donor vector for knocking membrane-tethered EGFP into the *PAX6* locus, the H2B::GFP sequence in the PAX6 H2B::GFP donor plasmid (Tchieu et al., 2017) (Addgene 105239, a kind gift from Lorenz Studer) was replaced by the sequence encoding a fusion of the mouse Ig Kappa chain V-III (for extracellular trafficking), EGFP, and PDGFRb transmembrane domain as a GFP ligand (Morsut et al., 2016) (Addgene 162223). Plasmids were designed with Snapgene and constructed using HiFi DNA assembly mix (E2621, NEB). Clones of the resultant PAX6:GFPL donor vector were verified by Sanger sequencing prior to use. HEK293T cells were transfected with PAX6:GFPL along with plasmids encoding the PAX6 Left and Right TALENs (Tchieu et al., 2017) (Addgene 109034 & 105525, a kind gift from Lorenz Studer) as reported. After puromycin (40 μg/mL) selection, cell colonies were picked under fluorescence microscopy. Targeted integration was confirmed by junction PCR using Q5 master mix (NEB). The following primer sequences were used for junction PCR to confirm GFP-L knock-in to the *PAX6* locus: 1-F 5′-GGG​TCA​TAG​GGT​TCC​CAA​AT-3′ and 1-R 5′- GAC​GTG​AAG​AAT​GTG​CGA​GA-3′, 2-F 5′-GGG​AGG​ATT​GGG​AAG​ACA​AT-3′ and 2-R 5′- GTG​GGT​ATA​AAT​GGG​CAC​AGA-3′. HEK293T and derived cells were cultured in DMEM-High Glucose with GlutaMAX (Gibco, 10569010), 10% heat-inactivated FBS (Gibco, 26140079), and 1% penicillin and streptomycin (Gibco, 15140122). The HEK293T-GFPL line was maintained under 40 μg/mL puromycin with medium changed every 2–3 days.

#### 2.1.2 Generation of “all-in-one” knock-in cell line of HEK293 inducible synNotch receiver cells

We targeted all transcriptional units required for synNotch receiver cell activity to the AAVS1-T2 site in HEK293 cells (ATCC). We cloned the synNotch receptor and the “payload” transgene expression cassettes into an all-in-one AAVSI-targeting plasmid (Mandegar et al., 2016) (Addgene 73497, a kind gift from Bruce Conklin). Thus, two expression cassettes were cloned between the AAVSI homology arms, resulting in a new plasmid deposited as Addgene 220238. One cassette permitted CAG promoter-driven expression of the synNotch receptor sensitive to GFP via use of a LaG17 nanobody (Fridy et al., 2014; Morsut et al., 2016). The second cassette enabled inducible TRE promoter-driven expression of both the morphogen Sonic Hedgehog and the fluorescent reporter mCherry, which were separated by an internal ribosome entry site (IRES). Further, a neomycin resistance cassette was introduced for cell selection.

The sequence of the gRNA used to target the AAVS1-T2 locus is 5′-GGG​GCC​ACT​AGG​GAC​AGG​AT-3′ (Synthego), as previously reported (Mali et al., 2013). HEK293 cells were transfected with the synNotch-encoding AAVSI targeting vector, HiFi Cas9 protein (1081060, IDT), and gRNA using the TransIT-X2 transfection kit (Mirus). The gRNA: HiFiCas9 ratio of 2:1 was used for transfection. After Geneticin (400 μg/mL) selection, cell colonies were picked under fluorescence microscopy. Targeted integration was confirmed by junction PCR using Q5 master mix (NEB). The primer sequences used for junction PCR to confirm synNotch knock-in to the AAVS1 locus were F 5′- TCG​TCC​TGC​AGT​TCA​TTC​AG-3′ and R 5′- CCA​GCT​CCC​ATA​GCT​CAG​TC -3′. The HEK293-synNotch knock-in cell line was maintained in 400 μg/mL geneticin.

### 2.2 SynNotch activation by anti-c-Myc beads

To determine whether an “all-in-one” synNotch knock-in would lead to the generation of functional HEK293 receiver cells, a series of anti-c-Myc tag magnetic beads (1, 2, or 3 µL corresponding to 10, 20, or 30 µg of beads, 88843 Thermo) were added to HEK293 synNotch knock-in cells cultured in a 24-well plate. Prior to adding the magnetic beads to cells, the beads were diluted in PBS and washed prior to resuspension in culture medium and supplementation to cell culture vessels. Non-cognate, anti-HA tag magnetic beads (3 µL or 30 μg, 88837 Themo) were similarly applied to cells as a control treatment. Three days after bead activation, HEK293 LaG17-syNotch cells were imaged for mCherry expression and further analyzed by flow cytometry.

### 2.3 CRISPRoff design and cell line generation

We previously reported a three component, doxycycline-inducible CRISPRoff system encoded in Sleeping Beauty transposon vectors (Shi et al., 2022; Gil et al., 2024). The first component of this platform is the transposon vector that encodes doxycycline-regulated expression of the CRISPRoff enzyme, which consists of a fusion of Dnmt3A, Dnmt3L, and the ZNF10 KRAB protein domains to dCas9 and blue fluorescent protein (BFP) (deposited at Addgene as plasmid #203355). A second transposon vector encodes cloning sites for U6-driven expression of sgRNAs specific to target genes of interest (deposited at Addgene as plasmid #203359). The third component involves the Sleeping Beauty 100x transposase plasmid (Mates et al., 2009) (Addgene # 34879, a kind gift from Zsuzsanna Izsvak), which provides a non-viral means of integrating the transposons into genomic DNA for stable expression.

Three PAX6-specific sgRNAs were cloned into the Sleeping Beauty transposon sgRNA vector. The following sequences were used: 5′-GAG​TGA​GAG​ATA​AAG​AGT​GT -3′; 5′-GAT​GTT​GCG​GAG​TGA​TTA​GT -3′; 5′- GTC​TCC​CGG​CGT​AGC​AGT​GG -3′, which were designed based on a published CRISPRi library (Horlbeck et al., 2016). A previously published, non-targeting (NT) sgRNA was used as a control (Thakore et al., 2015). Transposons encoding the sgRNAs (either the NT alone or the three PAX6-specific sgRNAs pooled) as well as the inducible CRISPRoff vector were co-transfected into PAX6::GFPL (GFPL) sender cells along with a plasmid encoding the Sleeping Beauty 100x transposase using TransIT-X2 (Mirus). Thus, the NT CRISPRoff and PAX6 CRISPRoff cell lines are derivatives of the GFPL sender cell line. After hygromycin (200 μg/mL, Invitrogen) and puromycin (40 μg/mL) selection, cells were supplemented with or without 1.5 μg/mL of doxycycline for 3 days. Samples were then sorted with a BD FACSAria III based on BFP expression. Sorted CRISPRoff sender cells were maintained under 40 μg/mL puromycin and 200 μg/mL hygromycin.

### 2.4 Cell co-cultures

#### 2.4.1 Co-culture of HEK293T-GFPL and HEK293-synNotch cells

We tested the activation of synNotch in cell lines by PAX6-driven GFP ligand with or without CRISPRoff perturbation. Sender cells (GFPL cells with or without CRISPRoff) were dissociated by TrypLE and washed at least twice with DPBS. Sender cells were then mixed with receiver HEK293 synNotch cells and co-cultured in DMEM-High Glucose with GlutaMAX (Gibco), 10% heat-inactivated FBS (Gibco), and 1% Pen/Strep (Gibco) without any other antibiotics. Three days later, two-dimensional (2D) co-cultures were imaged with an epifluorescence microscope (Leica Dmi8) and further analyzed via flow cytometry (CellStream, Luminex). For three-dimensional (3D) co-culture studies, mixtures of sender cells (either control 293T cells, NT CRISPRoff, PAX6 CRISPRoff) with HEK293 synNotch receiver cells were washed at least twice and pipetted in a round-bottom, 96-well culture plate (Costar, 7007). The plate was centrifuged for 5 min at 300x g before being placed into the incubator. For 3D co-cultures, samples were prepared for confocal microscopy (Nikon, Spinning Disk) 2 days after plating cells.

#### 2.4.2 Co-culture of HEK293T-GFPL and H9-synNotch-Ngn2 ESCs

The H9 human embryonic stem cell (hESC) line encoding LaG16-synNotch-driven expression of the master transcription factor Ngn2 was previously reported (Lee et al., 2023). To explore the neural differentiation of pluripotent stem cells through Ngn2 transgenic expression by synNotch activation, HEK293T-GFPL sender cells with or without CRISPRoff were dissociated by TrypLE and washed at least twice with DPBS. Sender cells were then mixed with the H9-synNotch-Ngn2 hESCs, which were dissociated with Accutase (07920, Stemcell Technologies). Mixed cells were plated in mTeSR Plus medium (1000274, Stemcell Technologies) in wells treated with Geltrex (Thermofisher) with an addition of 10 μM Y-27632 dihydrochloride, a ROCK inhibitor (Tocris). The medium was replaced daily with 5 μM Y-27632 dihydrochloride in mTeSR Plus. Three days after co-culture, cells were imaged using Leica Dmi8 epifluorescence microscope and further analyzed by Cytek Aurora flow cytometry (Cytek Biosciences). To detect expression of the neural marker TUJ1 induced by synNotch-driven hESC differentiation, 293T, NT CRISPRoff, and PAX6 CRISPRoff cells were washed and seeded respectively in mTeSR Plus medium on a Geltrex-treated plate. 2 days later, H9-synNotch-Ngn2 hESCs were added to 293T, NT CRISPRoff, and PAX6 CRISPRoff cells, separately. Three days later, cells were processed for immunofluorescence imaging.

### 2.5 Flow cytometry

Cells were dissociated into a single cell suspension with TrypLE (12604013, ThermoFisher) for HEK293T and HEK293, and their derived cells. For engineered H9-synNotch-Ngn2 hESCs and its mixture, cells were dissociated with Accutase (07920, Stemcell Technologies). Cells were then spun down at 300xg and resuspended in blocking buffer (1%FBS in DPBS). Single cell suspension samples and 10,000 cell events were run through the CellStream (Luminex), Cytek Aurora (Cytek Biosciences), or FORTESSA (BD Biosciences) analytical flow cytometer systems. Results were analyzed in FlowJo.

### 2.6 Immunofluorescence

To measure levels of PAX6 protein and reporter fluorescence from GFPL cells, samples were fixed in 4% paraformaldehyde in PBS for 10 min. After a DPBS wash, cells were permeabilized and blocked in 0.3% Triton-X (EMD Millipore) and 5% FBS in PBS for 10 min. Cells were then stained with an anti-PAX6 rabbit antibody (1:1000, 901301, Biolegend) and GFP-booster Alexa Fluor 488 (1:1000, gb2AF488, Chromotek) diluted in permeabilization and blocking buffer for 1 h at room temperature. After 3 times wash with DPBS, cells were then incubated with anti-rabbit IgG conjugated with Alexa-Fluor 594 (8889s, Cell Signaling) for 30 min. In a subset of experiments, samples were washed with DPBS and then counterstained with 4′,6-diamidino-2-phenylindoldihydrochloride (DAPI, 1 μg/mL, Thermo Scientific). Images were taken on a Leica Dmi8 epifluorescence microscope.

To detect hESC-induced differentiation by synNotch activation, cell mixtures of H9-synNotch-Ngn2 and 293T derived cells were fixed in 2% paraformaldehyde in PBS for 10 min. After DPBS washing, cells were permeabilized and blocked in PBS with 0.3% Triton-X (EMD Millipore) and 5% FBS (Gibco) for 10 min. Cells were then stained with anti-TUJ1 antibody conjugated to Alexa-Fluor 647 (1:500, 801210, Biolegend) and GFP booster (1:1000, gb2AF488, Chromo Tek) diluted in permeabilization and blocking buffer for 1 h. After washing, cells were imaged on a Leica Dmi8 epifluorescence microscope.

Image quantification was performed using ImageJ using default settings for mean fluorescence intensity and integrated density. Relative values were normalized to values measured from samples in which HEK293-synNotch receiver cells were co-cultured with HEK293T cells that were not engineered to express GFPL.

### 2.7 Statistical analysis

All bar graphs display means of at least triplicates, with replicate number indicated by individual points displayed. Error bars show standard error of the mean (SEM). For experiments involving only two comparisons, statistical significance was determined with a two-tailed unpaired *t*-test with alpha set to 0.05. To determine significance in experiments involving >2 groups or categorical variables, one-way ANOVA followed by Tukey’s *post hoc* test or two-way ANOVA followed by Bonferroni test were applied as appropriate with alpha set at 0.05. GraphPad Prism (Version 10) was used for statistical analysis.

## 3 Results

### 3.1 Characterization of GFPL knock-in sender cell line and an “all-in-one” AAVS1 knock-in cell line for mCherry inducible receiver cells

**FIGURE 1:**
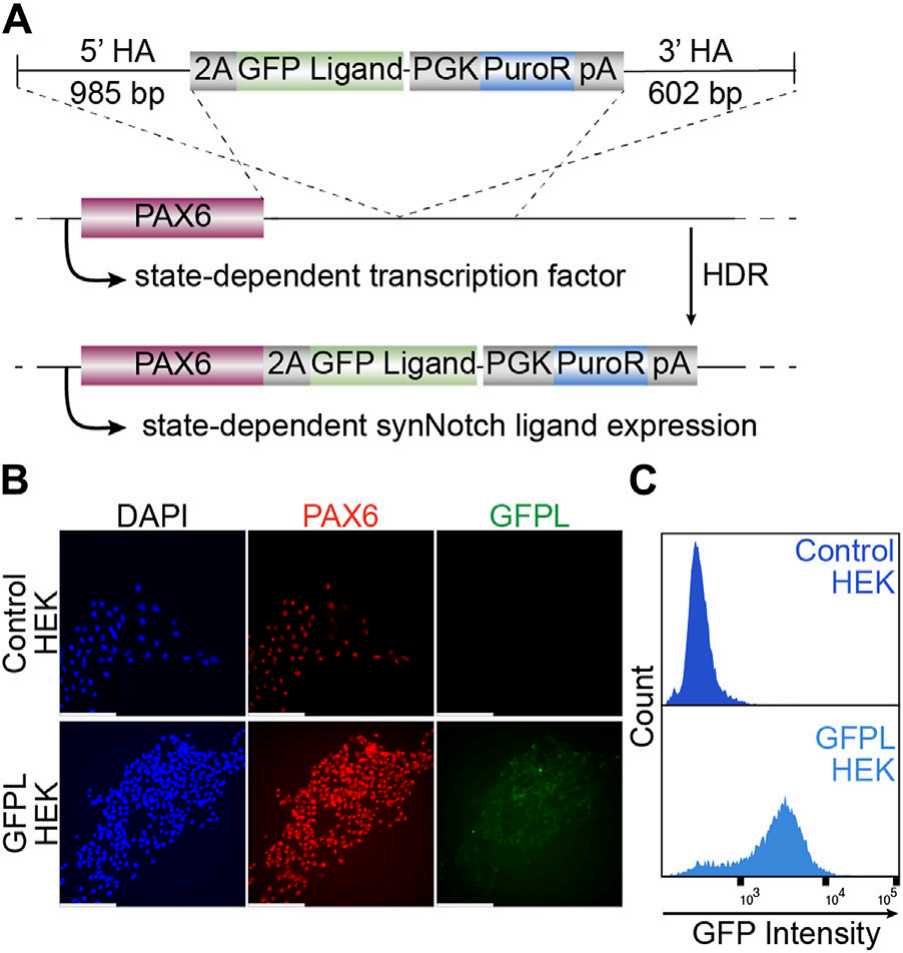
Generation of HEK293T line bearing a GFP ligand by gene targeting to the *PAX6* locus. **(A)** Schematic illustration of GFP ligand knock-in construct. 2A: ribosomal skipping peptide sequence; PGK: phosphoglycerate kinase promoter; PuroR: puromycin resistant transgene puromycin acetyltransferase; pA: polyadenylation signal. **(B)** Fluorescence microscopy of immunolabeled PAX6 (red), GFP ligand (green, labeled by GFP booster), and nucleus (DAPI, blue). Scale bar = 200 µm. **(C)** The GFPL-positive cells in PAX6:GFPL KI assessed by flow cytometry.

Surface expressed GFP, which consists of EGFP mounted onto a truncated PDGFRb transmembrane domain (Morsut et al., 2016) was integrated to the *PAX6* locus using TALEN-induced homology-directed repair. After puromycin selection and clonal isolation, knock-in cells were confirmed by genomic PCR of the junctions of the transgene and target locus (Supplementary Figure S1A). Then, we evaluated whether targeted transgene integration at the *PAX6* locus would enable EGFP transgene expression in HEK293T cells (Figure 1A). As shown via immunofluorescence (Figure 1B) and live cell imaging (Supplementary Figure S1C), EGFP protein is expressed at the membrane surfaces of cells. Meanwhile, PAX6 expression was also detected in the nuclei of the same cells, indicating the expression of membrane tethered-GFP expression is controlled by native PAX6. Further, flow cytometry detected the vast majority of GFPL knock-in cells as GFP positive, suggesting a homogeneous population of knock-in cells (Figure 1C).

**FIGURE 2:**
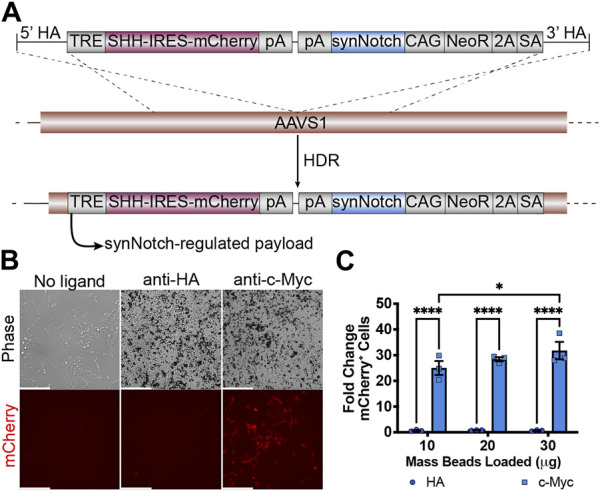
Generation of HEK293 line bearing a LaG17 (GFP nanobody) synNotch in the AAVS1 locus via CRISPR-Cas9 based genome editing. **(A)** Schematic illustration of synNotch knock-in construct. Note the receptor is N-terminally tagged with a c-Myc epitope. TRE: tetracycline response element; SHH: Sonic Hedgehog coexpressed with mCherry via an Internal Ribosome Entry Site (IRES); pA polyadenylation signal; CAG: CMV immediate enhancer/chicken β-actin promoter with a splice acceptor of the rabbit β-globin gene; NeoR: neomycin resistance transgene; 2A: ribosomal skipping peptide; SA: splice acceptor. **(B)** Fluorescence microscopy of 293 synNotch KI activated by anti-c-Myc beads, but not control anti-HA beads. Scale bar = 200 µm. **(C)** Flow cytometry quantification of bead-activated synNotch represented by mCherry signal. Results display the fold change in the fraction of mCherry-positive cells (normalized to synNotch cells cultured without beads). mean ± SEM, HA vs. c-Myc, *p < 0.05, ****p < 0.0001 by two-way ANOVA with Bonferroni *post hoc* test.

We then evaluated whether it is feasible to integrate all necessary transcriptional units required for receiver cell activity to one AAVS1 safe harbor site. A 12 kb plasmid including left and right homology arms was transfected to HEK293 cells together with AAVS1-T2 site-specific gRNA and Cas9 protein (Figure 2A). After puromycin selection and clonal isolation, knock-in cells were confirmed by junction PCR specific to the transgene and target locus (Supplementary Figure S1B). To determine whether the “all-in-one” knock-in generates functional receiver cells, anti-c-Myc beads were added to HEK293-synNotch cells. The synNotch receptor integrated into the AAVSI locus was engineered with an N-terminal c-Myc epitope tag, enabling identification of synNotch-positive cells by immunolabeling. This feature also potentiates activation of synNotch cells via beads coated with anti-c-Myc antibodies, which serve as “surrogate” ligands for c-Myc-tagged synNotch receptors. Thus, as expected, a dose-dependent mCherry expression was observed after anti-c-Myc bead stimulation, as assessed via both fluorescence microscopy (Figure 2B) and flow cytometry (Figure 2C). However, non-cognate anti-HA beads failed to induce mCherry expression, suggesting that a ligand-specific mechanical force (in this case, the binding between c-Myc tag and anti-c-Myc beads) is required to activate payload transgene expression from HEK293-synNotch receiver cells.

### 3.2 GFP ligand production under native *PAX6* control is sufficient to activate synNotch, yielding signal transduction in knock-in receiver cells

**FIGURE 3:**
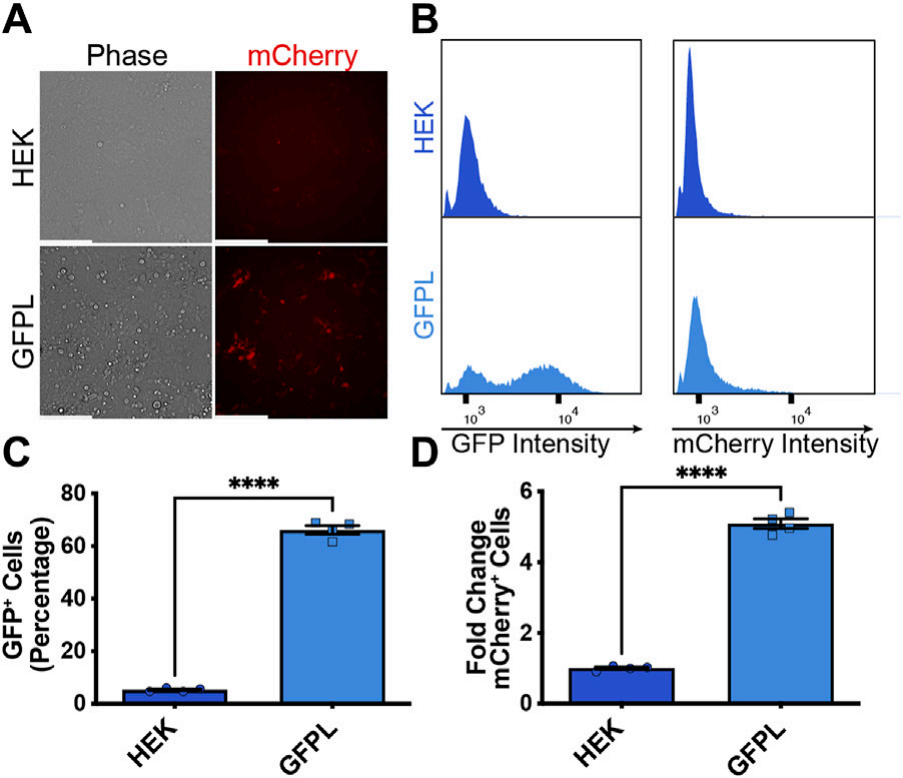
Activation of knock-in synNotch receiver cells by PAX6:GFP ligand (GFPL) cells. **(A)** Fluorescence microscopy of 293 synNotch KI cells activated by HEK293T line bearing GFPL, but not control 293T cells. Scale bar = 200 µm. **(B)** The GFPL-positive and mCherry-positive cells in a co-culture of the receiver cells (293 synNotch KI) and the sender cell line respectively (HEK or GFPL) assessed by flow cytometry. **(C, D)** Flow cytometry quantification of GFP-positive cells **(C)** and fold change in synNotch activation **(D)** in a co-culture of the receiver cells (293 synNotch KI) and either the HEK293T or GFPL sender cell line, respectively. Results display the fold change in the fraction of mCherry-positive cells (normalized to synNotch cells cultured with ligand-free 293T cells). mean ± SEM, ****p < 0.0001 by two-tailed unpaired *t*-test.

After demonstrating inducibility of the AAVSI-integrated synNotch gene circuit, we sought to test whether extracellular membrane-tethered EGFP under the control of endogenous *PAX6* transcription in sender cells is capable of inducing contact-mediated reporter expression in receiver cells. Thus, we combined AAVSI-synNotch receiver cells with GFPL sender cells and monitored mCherry expression. When co-cultured with GFPL knock-in cells, mCherry production was stimulated in HEK293-synNotch cells (Figure 3A). However, co-culture of AAVSI-synNotch cells with parental HEK293T cells (i.e., GFPL-deficient HEK293T cells) resulted in limited activation of HEK293-synNotch cells. We quantified the level of synNotch activation via flow cytometry. Results confirm that GFPL cells retain GFP expression (Figures 3B,C) adequate to drive significant mCherry upregulation in synNotch receiver cells (Figures 3B,D; Supplementary Figure S2). These results confirm the sufficiency of *PAX6*-regulated GFPL to activate expression of synNotch gene circuits expressed from the AAVSI locus of engineered receiver cells.

### 3.3 CRISPRoff perturbation of PAX6 levels propagates to synNotch receivers as reduced signal input

#### 3.3.1 *PAX6*-targeted CRISPRoff activity reduces AAVSI-HEK293 synNotch activation levels

**FIGURE 4:**
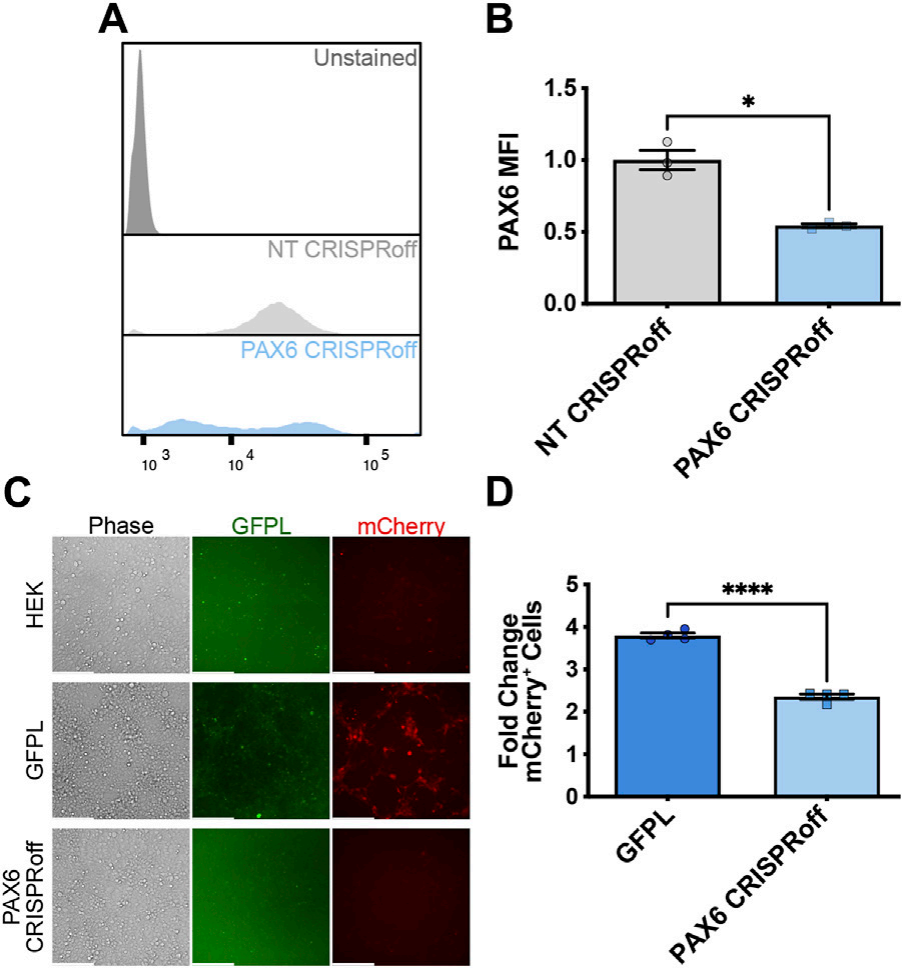
Reduction of synNotch activation in 2D culture by suppression of *PAX6* via CRISPRoff. **(A)** Representative flow cytometry histograms evaluating PAX6 immunofluorescence in GFPL cells treated with non-targeting (NT) or PAX6-specific CRISPRoff sgRNAs. **(B)** Quantification of the median fluorescence intensity of PAX6 levels in GFPL cells treated with NT or PAX6 CRISPRoff. mean ± SEM, *p < 0.05 by two-tailed unpaired *t*-test. **(C)** Fluorescence microscopy of a 2D co-culture of the receiver cells (293 synNotch KI) and the sender cell line HEK, GFPL, and PAX6 CRISPRoff, respectively. Scale bar = 200 µm. **(D)** Fold change of mCherry activation levels in 2D co-culture as assessed by flow cytometry. Results display the fold change in the fraction of mCherry-positive cells (normalized to synNotch cells cultured with ligand-free 293T cells). mean ± SEM, ****p < 0.0001 by two-tailed unpaired *t*-test.

Having established that natively expressed, *PAX6*-driven levels of GFPL can productively activate synNotch gene circuits integrated into AAVSI, we next sought to determine whether CRISPR-based manipulation of PAX6 levels would result in a corresponding alteration of synNotch signal activation in receiver cells. Thus, we deployed CRISPRoff to repress native *PAX6* expression in the GFPL HEK293T line. CRISPRoff provides a means to stably silence target genes because of heritably deposited DNA methylation at target loci (Nuñez et al., 2021). CRISPRoff repression occurs due to the combined activity of two *de novo* DNA methyltransferases (Dnmt3a and Dnmt3l) together with the KRAB repression module. These proteins are appended to catalytically inactive Cas9 (dCas9), which is further fused to blue fluorescent protein (BFP). Unlike traditional dCas9-KRAB CRISPRi systems, which induce heterochromatin formation at targeted chromosomal loci that is reversible once dCas9-KRAB expression decays, CRISPRoff enzymatically establishes DNA methylation and histone modifications that persist in the absence of durable expression of CRISPRoff protein (Nuñez et al., 2021). In our studies, expression of the Dnmt3a/l-dCas9-KRAB-BFP fusion protein was regulated by the doxycycline (dox)-inducible expression system. We engineered two CRISPRoff derivatives of the GFPL cell line: one expressing control, non-targeting (NT) sgRNAs, with the other expressing *PAX6*-specific sgRNAs (Horlbeck et al., 2016). We confirmed CRISPRoff expression via flow cytometry (Supplementary Figure S3). We validated that CRISPRoff cells transfected with *PAX6* sgRNAs (PAX6 CRISPRoff) showed significant reduction in PAX6 protein levels (Figures 4A,B), and demonstrated a corresponding reduction in GFPL expression (Supplementary Figure S4).

**FIGURE 5:**
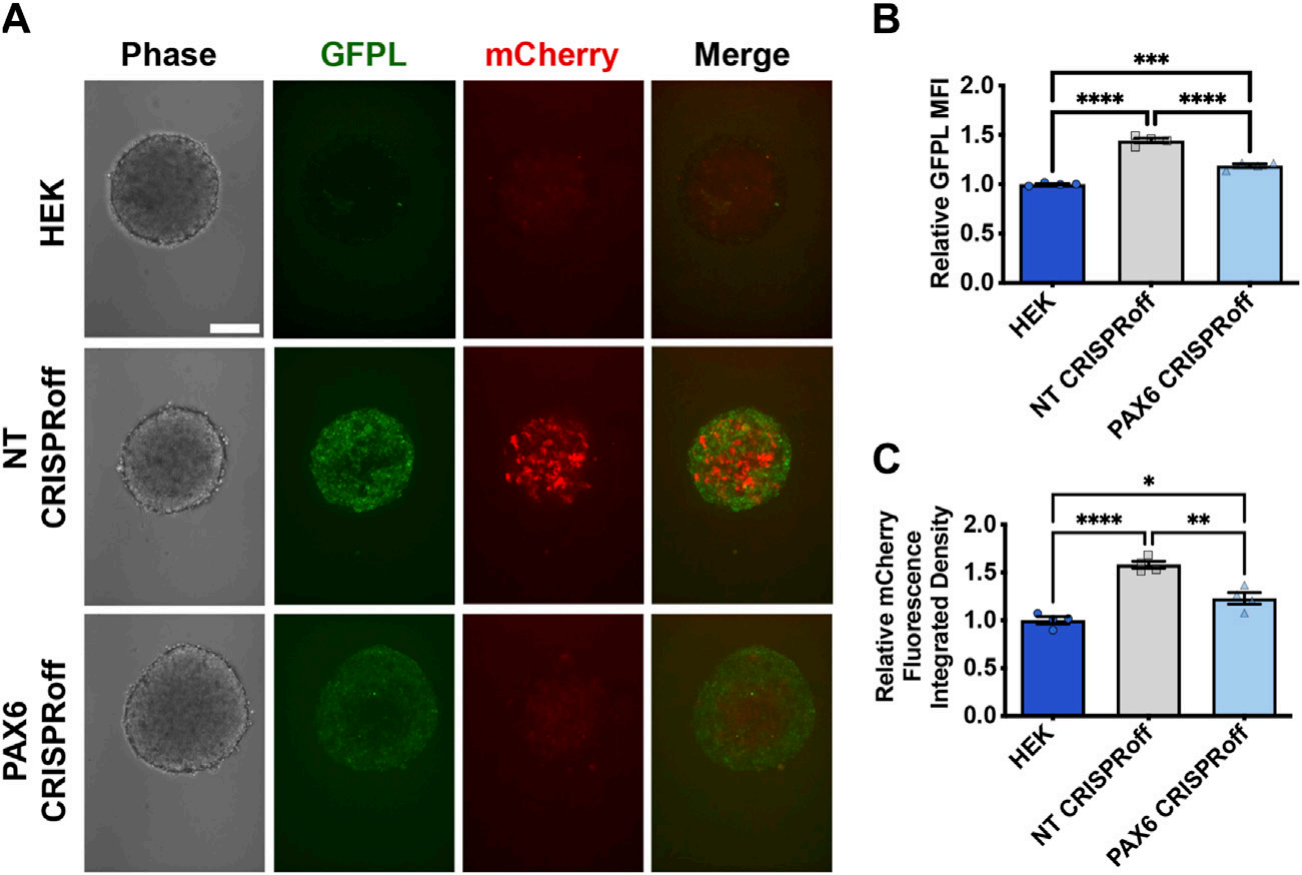
Reduction of synNotch activation in 3D culture by suppression of *PAX6* via CRISPRoff. **(A)** Confocal microscopy of 3D co-culture of the receiver cells (293 synNotch KI) and the sender cell line respectively (HEK, non-targeting (NT) CRISPRoff, or *PAX6*-specific PAX6 CRISPRoff). SynNotch activation is indicated by mCherry signal (red), and GFPL is visible in the green channel. Scale bar = 200 µm. **(B, C)** Quantification of GFPL intensity **(B)** and mCherry integrated density **(C)** in a 3D co-culture of the receiver cells (293 synNotch KI) with HEK, NT CRISPRoff, or PAX6 CRISPRoff sender cell lines respectively. Data was normalized to 293 synNotch KI cells co-cultured with ligand-free 293T cells. mean ± SEM, *p < 0.05, **p < 0.01, ***p < 0.001, and ****p < 0.0001 by one-way ANOVA with Tukey *post hoc* test.

We then proceeded with co-culture experiments to determine whether CRISPR-modulated PAX6 levels resulted in attenuated signal transmission to synNotch receiver cells. Thus, distinct sender cell populations (control, GFP-free HEK293T cells; GFPL cells; PAX6 CRISPRoff cells) were co-cultured with the AAVSI-synNotch receiver HEK293 cells. Indeed, we observed that CRISPRoff-based fine-tuning of PAX6 levels are transmitted as reduced synNotch activation (Figures 4C,D) in a 2D co-culture system. We then transitioned these co-culture conditions to 3D, spheroid co-culture models, reminiscent of synthetic morphogenesis studies (Toda et al., 2018; Glykofrydis et al., 2021). Again, we observed GFPL-dependent synNotch activation, which was significantly modulated upon CRISPRoff-mediated suppression of *PAX6* (Figure 5A). Consistent with our observations in the 2D culture system, image quantification revealed reduced GFPL levels in cells treated with PAX6 CRISPRoff cells (Figure 5B). The reduced levels of GFPL in turn led to a quantifiable reduction in the level of mCherry activation in synNotch receiver cells (Figure 5C). These results demonstrate a new approach for controlling artificial ligand presentation for synthetic signaling in engineered cell systems. Through this approach, ligand content activates synNotch to establish transcriptional levels of transgene reporters in receiver cells that mirror alterations of state changes in neighboring cells.

#### 3.3.2 Human embryonic stem cell fate selection dictated by CRISPR-regulated transcriptional states of neighboring cells

**FIGURE 6:**
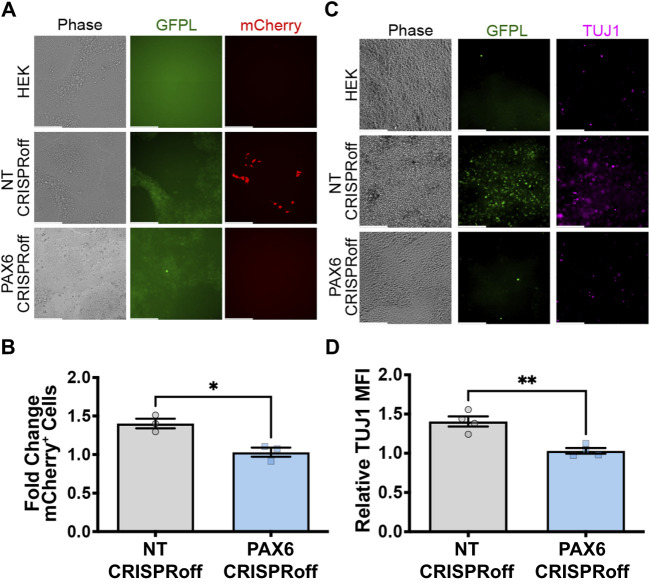
GFPL modulation determines neural differentiation of human pluripotent stem cells via synNotch signaling. **(A)** Fluorescence microscopy of co-cultures of the receiver cells (H9-synNotch-Ngn2, an hESC cell line engineered to inducibly express the master regulator of neurogenesis, Ngn2) and either a HEK, non-targeting (NT) CRISPRoff, or *PAX6*-specific PAX6 CRISPRoff sender cell line, respectively. SynNotch activation is indicated by mCherry signal in red, and GFPL is shown in green. **(B)** Flow cytometry quantification of synNotch activation in co-cultures. Results display the fold change in the fraction of mCherry-positive cells (normalized to H9-synNotch-Ngn2 cells co-cultured with ligand-free 293T cells). mean ± SEM, *p < 0.05 by two-tailed unpaired *t*-test. **(C)** Immunofluorescence images of co-culture of H9-synNotch-Ngn2 and each sender cell line showing the neurogenesis marker TUJ1 (purple). GFPL is stained by GFP booster (green). Scale bar = 200 µm. **(D)** Quantification of TUJ1 induction. Data were normalized by H9-synNotch-Ngn2 cells co-cultured with ligand-free 293T cells. mean ± SEM, **p < 0.01 by two-tailed unpaired *t*-test.

To explore whether GFP ligand linked to native *PAX6* expression can induce neural differentiation of pluripotent stem cells, we used an engineered H9 hESC that expresses mCherry and neurogenin-2 (Ngn2) in response to synNotch activation. Ngn2 is a master transcription factor capable of converting hESCs to TUJ1-positive motor neurons upon ectopic expression (Chavez et al., 2015; Lee et al., 2023). Thus, we co-cultured H9-synNotch-Ngn2 cells with HEK293-GFPL cells (either modified with non-targeting or specific PAX6 CRISPRoff sgRNAs). As expected, the NT CRISPRoff sender cells activate synNotch in H9 hESCs (Figures 6A,B). We also discovered that GFP-induced Ngn2 expression renders a TUJ1 positive population (Figures 6C,D) after co-culture of NT CRISPRoff with H9-synNotch cells. In those TUJ1 positive cells, neural projections were also displayed, suggesting a long-range connectivity among active neurons. However, GFP-free parental HEK293T cells and CRISPRoff cells with reduced PAX6 expression did not induce such mCherry expression or hESC differentiation (Figures 6A,C). These results illustrate that perturbation of endogenous *PAX6* expression in sender cells correspondingly impacts the fates of engineered H9-synNotch receiver cells.

## 4 Discussion

Synthetic biology tools, including CRISPR-based genetic/epigenetic modulation systems as well as artificial receptor modules, empower a build-to-understand approach to developmental systems. Artificial receptor platforms potentiate the forward engineering of cells with novel input-to-output linkages. This enables interrogation of the impact of dynamic, altered gene expression profiles on the allocation of cell phenotypes within a collective, heterogeneous cell population during development or in dysregulation associated with disease. Here, we demonstrate the ability to link an arbitrarily selected input, i.e., relative expression levels of PAX6 in sender cells, to user-selected outputs of mCherry reporter or Ngn2 transgene expression in synNotch receiver cells. We also demonstrate that varied PAX6 levels, modulated by CRISPRoff, result in attenuated relay of information via the synNotch signaling channel in engineered cells. In principle, we could have selected nearly any protein-coding locus to regulate GFPL levels. Similarly, we could exchange the mCherry/Ngn2 synNotch payloads for alternative transgenes. Thus, the principles underlying this approach allow for not only tracking of contact histories between cells, but also the prospective inquiry into interactive effects between perturbed gene regulation and the emergence of cell states of interest in human physiologic systems.

In HEK293T sender cells, knock-in of GFP ligand into the *PAX6* locus using nuclease-based targeted integration rendered sufficient expression of GFP ligand to activate synNotch signaling in receiver cells. To the best of our knowledge, this is the first time that expression of an artificial synNotch ligand has been coupled to a cell state marker such as PAX6 for receptor activation in human cells. Since our GFP ligand expression is linked to the transcription of *PAX6*, we were able to manipulate the production of GFPL through *PAX6* epigenetic modification by CRISPRoff. Indeed, CRISPRoff rendered a reduction in GFP ligand content in sender cells, which was faithfully mirrored by a reduction in synNotch activation levels in receiver cells. This design is compatible with both pharmacological and genetic/epigenetic manipulation of endogenous gene expression to regulate ligand levels in sender cells at physiologically relevant levels. For instance, one can control ligand production through the activation or suppression of its genetically coupled cell state marker by well-established CRISPR activation or more traditional CRISPR interference methods, in addition to the CRISPRoff manipulation demonstrated here (Stover et al., 2017; Jost et al., 2021; Nuñez et al., 2021). User selection between these technologies will depend on application (e.g., upregulation vs. suppression) and the need for durable (i.e., CRISPRoff) vs. easily reversible epigenetic modifications (i.e., CRISPRi; CRISPRa). Other considerations will include compatible targeting windows (compared to CRISPRi, CRISPRoff effectively silences target genes thanks to a broad targeting window within gene promoters) and specificity, which is similarly constrained across the technologies (Nuñez et al., 2021). Thus, this approach represents a powerful tool to further pinpoint roles of specific genes in cellular decision-making in the context of dynamically changing microenvironments consisting of epigenetically distinct cells.

In this work, we elected to knock the synNotch receptor and payload gene circuit into the AAVSI locus of HEK293 receiver cells. Our rationale for this approach is multifaceted. Use of this safe harbor locus helps to protect against transgene silencing, which persists as a major challenge for robust mammalian cell engineering (Okada and Yoneda, 2011; Cabrera et al., 2022). Further, nuclease-based knock-in of transgene cargos to chromosomal DNA avoids random integration, improves homogeneity of engineered cells, and can accommodate large DNA cargos as compared to viral transgene delivery (Kaufman et al., 2008; Perez-Pinera et al., 2012). The relative low copy integration events obtained with chromosomal knock-in (1–2) may result in a tradeoff of lower absolute levels of synNotch expression and subsequent payload transgene expression. By comparison, lentiviral delivery used in several studies of synNotch gene circuits (Morsut et al., 2016; Roybal et al., 2016; Toda et al., 2020), including our own (Lee et al., 2023), can lead to integration events averaging 5 vector copies per transduced cell (Woods et al., 2003). To contextualize this tradeoff, activation of the AAVSI-synNotch HEK293 cells via the potent stimulation of anti-c-Myc beads in this study resulted in ∼25–30x induction levels, whereas our previously published study involving lentiviral transduction (Lee et al., 2023) showed levels of activation of a similar GFP-responsive synNotch receptor of over 200x relative to untreated controls. On the other hand, excessive expression of synNotch receptors in cells may result in undesired ligand-independent activation to compromise the application (Yang et al., 2020; Sloas et al., 2023). Nevertheless, our results show that AAVSI-inserted synNotch circuits are competent to potently respond to GFPL expressed from the native *PAX6* locus, indicating a capacity to respond to low-copy insertions of synNotch ligand expressed downstream of a cell state-specific promoter.

An attractive feature of synthetic Notch is to provide flexibility in cellular responses with customized response behaviors to achieve combinatorial integration of user-defined environmental cues (Roybal et al., 2016; Toda et al., 2020). One of the most intriguing applications of synNotch is to trace and control stem cell fate decisions (Malaguti et al., 2022; Zhang et al., 2022; Lee et al., 2023). Here, we show that *PAX6*-driven expression of GFP ligand can induce the neural differentiation of H9 human embryonic stem cells through synNotch activation and its subsequently controlled Ngn2 transgene expression. Consistent with our prior work (Lee et al., 2023), synNotch induction rapidly caused conversion of H9 hESCs to TUJ1 positive cells. With these arbitrarily selected cell programs, we can toggle genes at any point during differentiation of stem cells to exploit developmental biology and tissue engineering. Furthermore, because multiple orthogonal synNotch receptors sensitive to distinct inputs can be deployed in individual cells, we can drive bifurcated responses based on alternative cell state changes adopted by neighbors undergoing morphogenesis. Our success in deploying this system to influence stem cell differentiation foreshadows an ability to extend this approach in the context of organoid production, where dynamic, morphogenetic interactions give rise to both self-organization and cell type diversification in manners that are both poorly understood and difficult to regulate. Though potentially transformative, one key limitation of this approach is the dependence on cell-cell contact. Several cellular interactions, such as paracrine signaling via morphogens, cytokines, extracellular vesicles, and other factors, can take place in the absence of direct cell-cell contact. Thus, the method we describe here is most applicable to contact-based histories and would not capture these additional interactions without considerable modification. Further, our approach requires extensive cell engineering, including targeted integration of the synNotch ligand to a relevant gene that serves as a cell state marker, and engineering of the cells to express the synNotch receptor and transgene payload. This work demonstrates that the application of synthetic biology tools in this space may facilitate an improved understanding of how pluripotent cells respond to one another in emerging organoids, or this technology may be used to instruct organogenesis for feedback-controlled, state-responsive transgene expression. Similarly, the technology can be expanded to scaffold-based tissue engineering strategies. Thus, this work provides a roadmap for how to combine advanced cell control technologies to interrogate and ultimately govern cell-cell interactions and to probe the influence of targeted changes on collective cell decision-making processes.

## Data Availability

The raw data supporting the conclusion of this article will be made available by the authors, without undue reservation.
