# Supplementary material for: Use of CRISPRoff and synthetic Notch to modulate and relay endogenous gene expression programs in engineered cells
Source: Front Bioeng Biotechnol. 2024 Jun 18;12:1346810. doi: 10.3389/fbioe.2024.1346810 (PMC11218679; doi:10.3389/fbioe.2024.1346810)
Supplement: Supplementary file 1 [file Image1.pdf]

**Supplemental Materials**

**Supplemental Figures:**

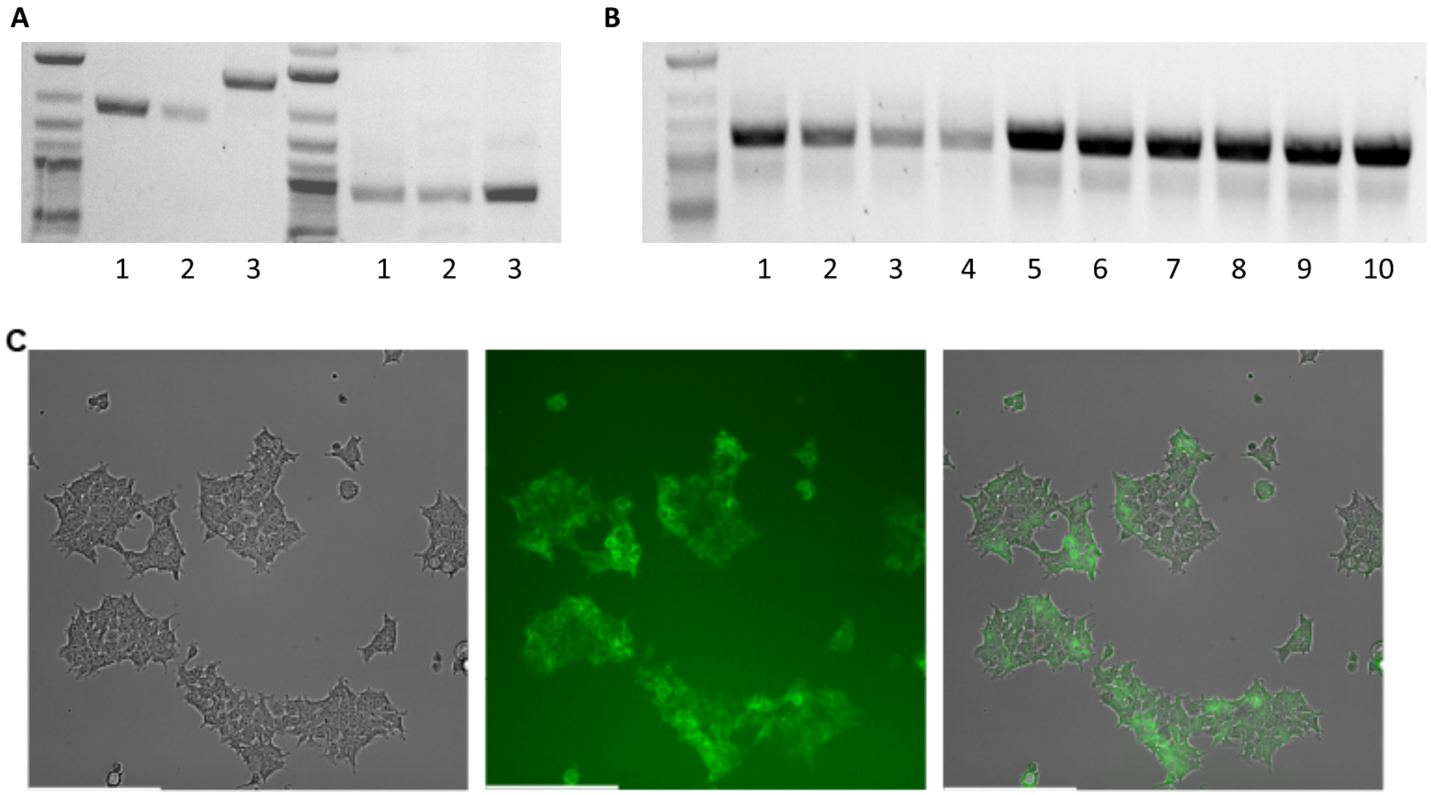


**Fig. S1. Genomic junction PCR of engineered cells and membrane expression of GFPL.** Related to Fig. 1. (A) Genomic junction PCR from HEK293T clones isolated after TALEN targeted integration and puromycin selection. Left: junction PCR for Left Homology Arm integration, right: Right Homology Arm integration. Lanes 1 & 2: GFP-H2B knock-in to the PAX6 locus, using the original donor vector as reported by Tchieu and colleagues (Tchieu et al., 2017), where GFP-H2B is the knock-in transgene. Lane 3: Knock-in of GFP ligand transgene to the PAX6 locus for the work presented here. (B) Genomic junction PCR from 10 HEK293 clones isolated after CRISPR-Cas9 targeted integration and G418 selection. SynNotch cassettes are knocked into the AAVS1-T2 locus (Mali et al., 2013). (C) Phase contrast and fluorescence microscopy (along with merged image) showing membrane-associated GFPL after knock-in of the synNotch GFP ligand to the *PAX6* locus in HEK293T cells. Scale bar = 200 µm. As compared to cells in Fig. 1B, these cells are not fixed or permeabilized.

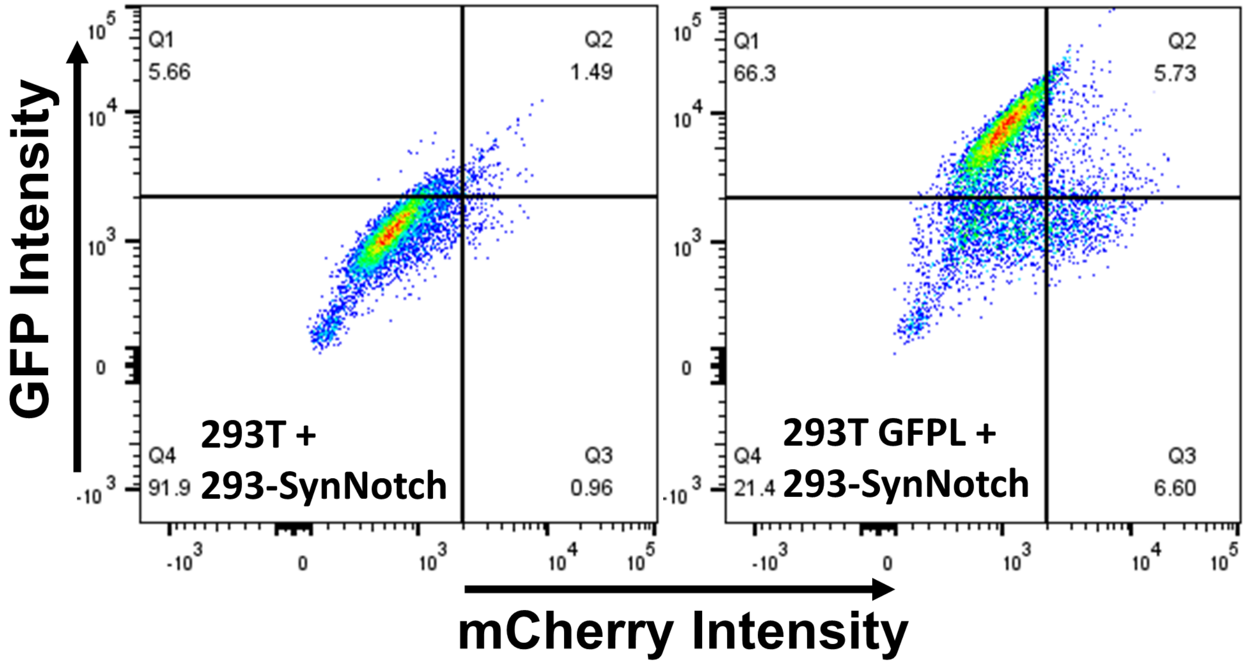


**Fig. S2. Dual-axis FACS dot plots related to Fig. 3B.** Cells in Fig. 3A-B were subjected to flow cytometry. On the left, a dot plot from a sample of HEK cells co-cultured with AAVSI-synNotch knock-in HEK cells capable of turning mCherry positive. On the right, a dot plot from a sample of GFP-L HEK cells co-cultured with the same synNotch knock-in HEK cell line. The result shows a distinct shift of a GFP+ population attributable to GFP-L cells in the dot plot on the right. There is also a separate, distinct population that exhibits increasing mCherry positivity, and this is exclusive to the co-culture involving both GFP-L and synNotch cells.

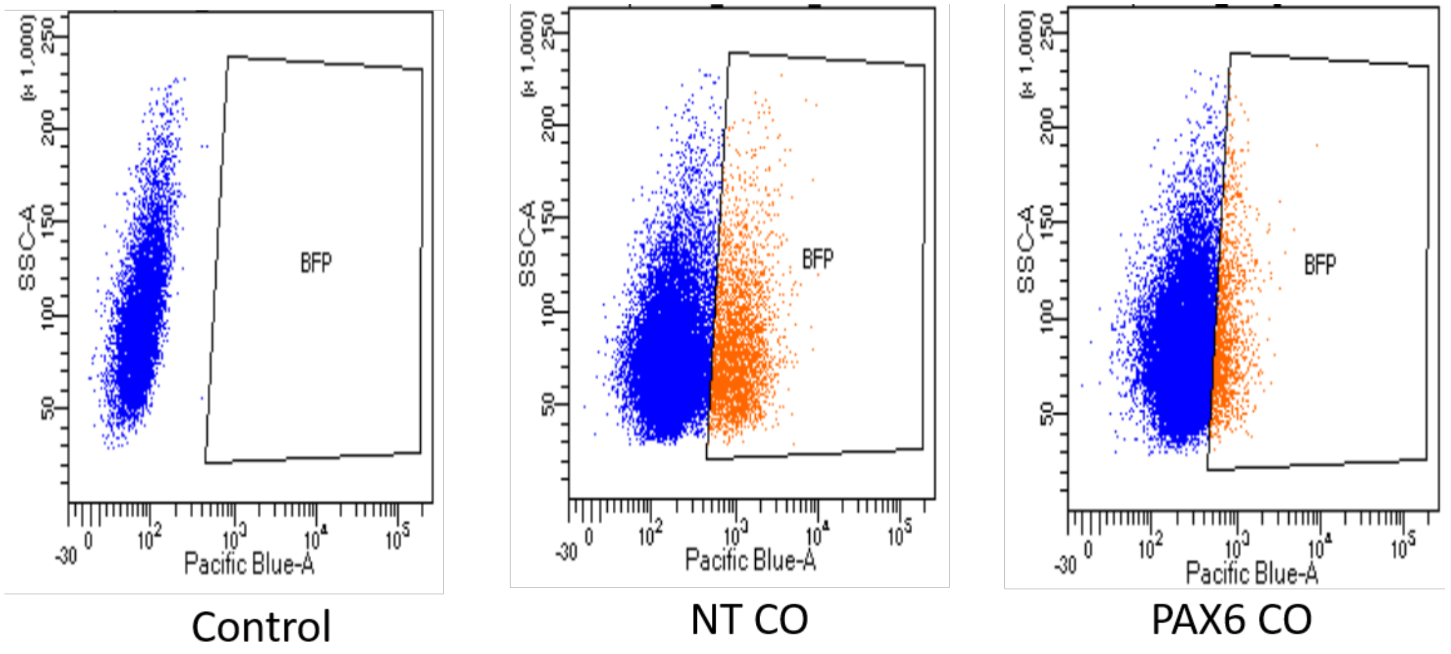


**Fig. S3: Doxycycline induced CRISPRoff expression indicated by BFP fluorescence in the engineered cell lines.** The CRISPRoff enzyme, which consists of a fusion of Dnmt3A, Dnmt3L, and the ZNF10 KRAB protein domains to dCas9 and blue fluorescent protein (BFP) was constructed into a single plasmid (Addgene, #203355, Gil et al., 2024). GFP-L cells engineered with CRISPRoff enzyme and non-targeting (NT CO) and PAX6 (PAX6 CO) single guide RNAs were sorted with the same gate.

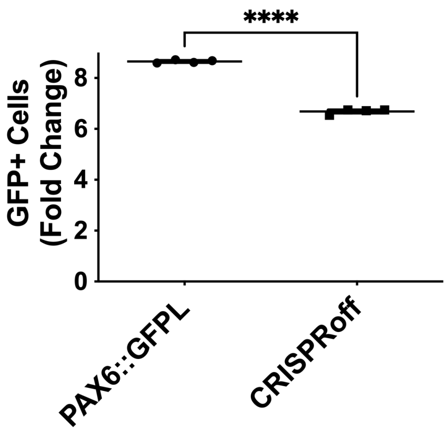


**Fig. S4. Reduction of GFP+ cells after CRISPRoff treatment.** Related to Fig. 4. The GFP+ cells in 2D co-culture were assessed by flow cytometry (normalized by ligand-free 293T and 293-synNotch KI cell co-cultures). mean ± SEM, **** $p < 0.0001$ by two-tailed unpaired T-test.

**References:**

Tchieu, J., Zimmer, B., Fattahi, F., Amin, S., Zeltner, N., Chen, S., & Studer, L. (2017). A Modular Platform for Differentiation of Human PSCs into All Major Ectodermal Lineages. *CELL STEM CELL*, *21*(3), 399+. doi:10.1016/j.stem.2017.08.015

Mali, P., Yang, L., Esvelt, K. M., Aach, J., Guell, M., DiCarlo, J. E., … Church, G. M. (2013). RNA-guided human genome engineering via Cas9. *Science (New York, N.Y.)*, *339*(6121), 823–826. doi:10.1126/science.1232033

Gil, M., Hamann, C.A., Brunger, J.M., Gama, V. (2024). Engineering a CRISPRoff Platform to Modulate Expression of Myeloid Cell Leukemia (MCL-1) in Committed Oligodendrocyte Neural Precursor Cells. Bio Protoc, 14(1):e4913. doi: 10.21769/BioProtoc.4913
